# Optimizing Spectronaut Search Parameters to Improve Data Quality with Minimal Proteome Coverage Reductions in DIA Analyses of Heterogeneous Samples

**DOI:** 10.1021/acs.jproteome.3c00671

**Published:** 2024-05-01

**Authors:** Christa P. Baker, Roland Bruderer, James Abbott, J. Simon C. Arthur, Alejandro J. Brenes

**Affiliations:** † Division of Cell Signalling & Immunology, School of Life Sciences, University of Dundee, Dundee DD1 5EH, United Kingdom; ‡ Biognosys AG, Schlieren, Zurich 8952, Switzerland; § Data Analysis Group, Division of Computational Biology, School of Life Sciences, University of Dundee, Dundee DD1 5EH, United Kingdom

**Keywords:** DIA, spectronaut, false positives, XIC, data quality, multispecies, *M. musculus*, C. albicans, infection, immunology

## Abstract

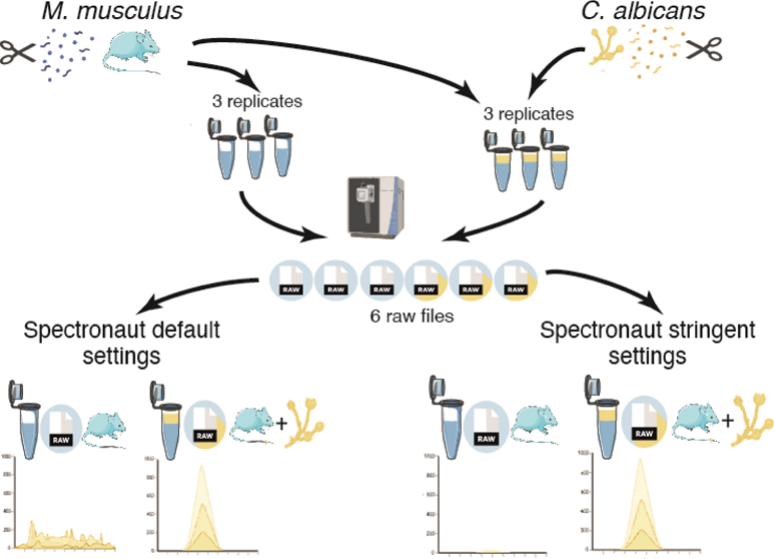

Data-independent acquisition has seen breakthroughs that enable comprehensive proteome profiling using short gradients. As the proteome coverage continues to increase, the quality of the data generated becomes much more relevant. Using Spectronaut, we show that the default search parameters can be easily optimized to minimize the occurrence of false positives across different samples. Using an immunological infection model system to demonstrate the impact of adjusting search settings, we analyzed *Mus musculus* macrophages and compared their proteome to macrophages spiked with*Candida albicans*. This experimental system enabled the identification of “false positives” as *Candida albicans* peptides and proteins should not be present in the *Mus musculus*-only samples. We show that adjusting the search parameters reduced “false positive” identifications by 89% at the peptide and protein level, thereby considerably increasing the quality of the data. We also show that these optimized parameters incurred a moderate cost, only reducing the overall number of “true positive” identifications across each biological replicate by <6.7% at both the peptide and protein level. We believe the value of our updated search parameters extends beyond a two-organism analysis and would be of great value to any DIA experiment analyzing heterogeneous populations of cell types or tissues.

## Introduction

Data-independent acquisition (DIA) has become a popular method to analyze label-free proteomes at scale and without a considerable compromise in depth.^1,2^ Unlike data-dependent acquisition (DDA), DIA does not select a subset of precursor ions to be fragmented for MS2 analysis; instead, it fragments all ions over a specific M/Z window.^3−5^ Consequently, the spectra derived from DIA data are more complex and convoluted than those derived from DDA. This initially meant that software tools^6−8^ required libraries to be generated, either spectra or peptide-centric, in order to analyze and match DIA data. Library generation became a barrier due to cost and time constraints; hence, the development of library-free DIA was a major advance that helped to popularize the method. Today, library-free single-shot DIA workflows are a standard technique for large-scale comprehensive proteomics analyses.

DIA can now achieve comparatively comprehensive proteome coverage while maintaining scalability,^1,2,9^ as such DIA-based analyses to study the immune system have also become more common and important.^10−18^ In this type of study, it is commonly necessary to analyze a host organism, frequently*Mus musculus* (*M. musculus*), a good model to study immunological networks which are broadly applicable to humans, and an invading pathogen, such as*Candida albicans* (C*. albicans*). This type of analysis introduces additional complications to the standard proteomic workflows. Analyzing biological samples that include more than one species increases the search space and introduces peptide mapping issues when there is sequence homology between the different peptidomes.^19^ To address this challenge, we explored the impact of different Spectronaut settings on the identification of false positives in samples with lysates derived from 1 or 2 species. Here, we analyzed the proteomes of *M. musculus* bone marrow-derived macrophages (BMDMs) in the presence or absence of the fungal pathogen*C. albicans*.

Macrophages are key cells of the innate immune system, which are known to help control infections and diseases, such as *C. albicans*(20−23). *C. albicans* is an opportunistic pathogen found in most healthy adults but can quickly turn into a difficult-to-treat systemic infection if the immune system is suppressed and can carry a 46–75% mortality rate.^21−23^ Macrophages are one of the first immune cells which respond to and kill *C. albicans*. They recognize *C. albicans* with pattern recognition receptors, resulting in phagocytosis of *C. albicans* yeast as well as macrophage activation. Activated macrophages produce an antifungal responce including reactive oxygen species production to aid killing, and inflammatory cytokines to attract other immune cells to the site of infection.^24−31^

Our results show that despite limited homology between the two species, a library-free workflow using directDIA with the default search settings incorrectly identified over 1,000 *C. albicans* peptides in the nonspiked BMDM samples. Within this study, we share optimized search parameters for Spectronaut that minimize the number of misidentifications, i.e., *C. albicans* peptides detected in the *M. musculus*-only samples, at a reduced penalty to overall proteome coverage. We believe our suggested settings will provide alternatives for more high stringency, robust identification results for library-free DIA for multispecies that are also applicable to the analysis of heterogeneous populations, be it different cell types or tissues.

## Experimental Section

### Animal and Cell Culture

All mouse work was done at the University of Dundee, following the UK Home Office license (PAAE38C7B) and approved by the Ethical Review and Welfare Committee at the University of Dundee. C57Bl6/J mice were obtained from Charles River Laboratories, UK. The animals were maintained in accordance with UK and EU regulations and were kept in a specific pathogen-free environment in individually ventilated cages. Nonbreeding mice were maintained in the same sex cage groups, with access to water and food (R&M1 SDS, Special Diet Services). Animals were kept in light and dark (12/12 h) cycle rooms at 21 °C with 45–65% humidity. Mice were culled using increased CO_2_ in cages, followed by confirmation of death by cervical dislocation.

### Murine Bone Marrow-Derived Macrophage Cell Culture

Bone marrow-derived macrophages (BMDMs) were cultured in L929 medium as detailed. On day 0, bone marrow was flushed with PBS from the femurs and tibias of a *M. musculus* using a 25G needle and 20 mL syringe onto a 90 mm bacteriological grade plate (Thermo Scientific, 101R20). Once flushed, the bone marrow was then filtered through a 100 μm strainer, into a 50 mL falcon tube, and centrifuged at 400*g* for 5 min. The bone marrow was then resuspended in 100 mL of L929 conditioned media (DMEM (FISHER Invitrogen, Cat. 11510416) and supplemented with 10% heat-inactivated FBS (Labtech), 10% week 1 L929 conditioned media, 10% week 2 L929 conditioned media, 500 mL 100 μg/mL penicillin and 100 μg/mL streptomycin (GiBCO Life Technologies), 1% sodium pyruvate (Lonza), 1% 1x nonessential amino acids (Lonza), 50 μM 2-mercaptoethanol, 1% GlutaMAX (GIBCO Life Technologies), and 1% HEPES (Lonza). Week 1 and week 2 L929 media were collected from L929 fibroblasts in the media recipe listed above but without the L929 media supplements. Cells were cultured for 7 days on bacterial grade plates (Thermo Scientific, 101R20) at 37 °C and 5% CO_2_. After 7 days, BMDMs were detached by scraping in PBS with 1% EDTA, counted using a hemocytometer, replated at 1 × 10^6^ cells in 2 mL of L929 media on a 6-well TC treated plate (Greiner, 657–160), and allowed to seed overnight. The following morning, cells were gently washed three times with PBS, and then cells were ready for lysis for proteomics.

### *C. albicans* Cell Culture

Frozen SC5314 *C. albicans* strain (20% glycine, 80% YPD media containing *C. albicans* yeast) was used to streak a YPD agar plate (YPD medium +2% agar) in sterile conditions and incubated overnight at 30 °C. After distinct colony growth, a single colony was picked using a pipet tip and cultured in 5 mL of YPD media broth overnight in 30 °C at 200 rpm.

### Lysis for Proteomics

For this experiment, 3 mice were used, and one colony was cultured from *C. albicans*. BMDMs and *C. albicans* were lysed separately using 400 μL/1 × 10^6^ cells with 5% SDS (20% SDS Sigma, 05030), 10 mm TCEP (0.5 M TCEP Thermo Fisher Scientific, 77720), 50 mM TEAB (1 M TEAB Thermo Fisher Scientific, 90114), and HiPerSolv Water for HPLC (VWR, 83650.320). Lysis and proteomic sample preparation are as described.^17^ Briefly, after lysis, lysates were boiled at 100 °C for 5 min and then sonicated before protein concentration was calculated using the EZQ protein quantification kit (Thermo Fisher Scientific, R33200). Each of the 3 *M. musculus* biological replicates were separated into two aliquots each containing 200 μg of protein. One aliquot contained only murine BMDMs (nonspiked), while the other aliquot contained BMDMs spiked with 50 μg of *C. albicans* protein (spiked). Tryptic peptides were generated by the S-Trap method using S-Strap: Rapid Universal MS Sample Prep (Co2 mini, Protifi) and Trypsin Gold (Promega, V5280). Samples were then vacuum-dried and resuspended in 50 μL of 1% formic acid (Thermo Fisher Scientific, 695076). Sample peptides were calculated via a CBQCA quantification kit (Thermo Fisher) and were then ready for MS analysis.

### Liquid Chromatography–Mass Spectrometry

Samples were analyzed on an Orbitrap Exploris 480 (Thermo Fisher) in DIA mode.^17^ For each sample, 1.5 μg of peptides was analyzed on the Exploris 480 coupled with a Dionex Ultimate 3000 RS (Thermo Scientific). LC buffers were prepared as follows: buffer A (0.1% formic acid in Milli-Q water (v/v)) and buffer B (80% acetonitrile and 0.1% formic acid in Milli-Q water (v/v)). 1.5 μg aliquots of each sample were loaded at 15 μL/min onto a trap column (100 μm × 2 cm, PepMap nanoViper C18 column, 5 μm, 100 Å, Thermo Scientific) equilibrated in 0.1% trifluoroacetic acid (TFA). The trap column was washed for 5 min at the same flow rate with 0.1% TFA and then switched in line with a Thermo Scientific, resolving the C18 column (75 μm × 50 cm, PepMap RSLC C18 column, 2 μm, 100 Å). The peptides were eluted from the column at a constant flow rate of 300 nL/min with a linear gradient from 3% buffer B to 6% buffer B in 5 min, then from 6% buffer B to 35% buffer B in 115 min, and finally to 80% buffer B within 7 min. The column was then washed with 80% buffer B for 6 min and re-equilibrated in 3% buffer B for 15 min. Two blanks were run between each sample to reduce carry-over. The column was kept at a constant temperature of 50 °C at all times.

The data were acquired using an easy spray source operated in positive mode with a spray voltage at 1.9 kV, a capillary temperature at 250 °C and a funnel RF at 60 °C. The MS was operated in data-independent acquisition (DIA) mode. A scan cycle comprised a full MS scan (*m*/*z* range from 350–1650, with a maximum ion injection time of 20 MS, a resolution of 120,000, and an automatic gain control (AGC) value of 5 × 10 ^6^). MS survey scan was followed by MS/MS DIA scan events using the following parameters: default charge state of 3, resolution of 30.000, maximum ion injection time of 55 MS, AGC of 3 × 10^6^, stepped normalized collision energy of 25.5, 27, and 30, and fixed first mass of 200 *m*/*z*. The inclusion list (DIA windows) and window widths are shown in Table 1. Data for both MS and MS/MS scans were acquired in profile mode. Mass accuracy was checked before the start of sample analysis.

**Table 1:** DIA Windows

window	window start (*m*/*z*)	window width	window overlap	window	*m*/*z*	isolation window	window overlap
1	349.975	66.8	0.525	**24**	663.25	14.5	0.5
2	416.25	13.5	0.5	**25**	677.25	13.5	0.5
3	429.25	11.5	0.5	**26**	690.25	13.5	0.5
4	440.25	12.5	0.5	**27**	703.25	14.5	0.5
5	452.25	11.5	0.5	**28**	717.25	16.5	0.5
6	463.25	11.5	0.5	**29**	733.25	15.5	0.5
7	474.25	11.5	0.5	**30**	748.25	16.5	0.5
8	485.25	10.5	0.5	**31**	764.25	18.5	0.5
9	495.25	11.5	0.5	**32**	782.25	17.5	0.5
10	506.25	11.5	0.5	**33**	799.25	18.5	0.5
11	517.25	11.5	0.5	**34**	817.25	19.5	0.5
12	528.25	10.5	0.5	**35**	836.25	20.5	0.5
13	538.25	11.5	0.5	**36**	856.25	20.5	0.5
14	549.25	10.5	0.5	**37**	876.25	22.5	0.5
15	559.25	11.5	0.5	**38**	898.25	24.5	0.5
16	570.25	10.5	0.5	**39**	922.25	26.5	0.5
17	580.25	11.5	0.5	**40**	948.25	28.5	0.5
18	591.25	12.5	0.5	**41**	976.25	31.5	0.5
19	603.25	12.5	0.5	**42**	1007.25	35.5	0.5
20	615.25	12.5	0.5	**43**	1042.25	41.5	0.5
21	627.25	11.5	0.5	**44**	1083.25	50.5	0.525
22	638.25	13.5	0.5	**45**	1133.225	516.8	
23	651.25	12.5	0.5				

### Instrument & Software Parameters

The specific instrument parameters are listed below (Table 2). The DIA-based mass spectrometry data were processed in Spectronaut 16 and 17. The default search parameters (Table 3), along with optimized more stringent parameters (Table 4), are also listed below.

**Table 2:** Instrument Parameters

**MS survey scan**	
spray source	positive mode
spray voltage	2.650 kV
ion transfer tube temperature	250 °C
MS1 orbitrap resolution	120,000
MS2 orbitrap resolution	30,000
full MS scan cycle	350–1650 *m*/*z*
RF lens (%)	40
AGC (%)	300
maximum injection time mode	custom
maximum injection time	20 ms
source fragmentation	disabled

**MS survey scan followed by MS/MS DIA Scan events**	
multiplex ions	false
collision energy mode	stepped
collision energy type	normalized
HCD collision energies (%)	25.5,27,30
orbitrap resolution	30,000
first mass	200
RF lens (%)	40
AGC target	custom
normalized AGC target (%)	3000
maximum injection time mode	custom
maximum injection time	55 ms

**Table 3:** Default Spectronaut Identification Settings

**default identification settings**	
precursor *q*-value cutoff	0.01
precursor PEP cutoff	0.2
Protein FDR strategy	accurate
protein *q*-value cutoff (experiment)	0.01
protein *q*-value cutoff (run)	0.05
protein PEP cutoff	0.75

**Table 4:** Stringent Spectronaut Identification Settings

**stringent identification settings**	
precursor *q*-value cutoff	0.01
precursor PEP cutoff	0.01
protein FDR strategy	accurate
protein *q*-value cutoff (experiment)	0.01
protein *q*-value cutoff (run)	0.01
protein PEP cutoff	0.01

### Data Normalization and Statistics

The median intensities were calculated for each sample after filtering out any proteins with an intensity of 0. The individual protein intensities across each sample were divided by the sample median. Volcano plots were made using −Log_10_ (*p*-value) and Log_2_ fold change (spiked/nonspiked) samples. The *p*-values and fold change values were calculated using the bioconductor package, 'Limma'^32^, after Log_2_ transforming the normalized intensity data. *Q*-values were calculated using the bioconductor package 'qvalue'. For the volcano plot, the −Log_10_ (*p*-values) versus the Log_2_ (fold change) were plotted, with a *p*-value of 0.01 denoted at a horizontal line and a fold change of −2,2, as two vertical lines. Proteins were considered to be significantly changed when the q-value<0.05 and the log_2_ fold change >1 or <−1. Significant proteins were highlighted in blue for downregulated proteins and red for upregulated proteins.

### Theoretical Tryptic Digest

Protein sequences were downloaded from UniProt in January, 2023. *Mus musculus* C57BL/J6 sequences, including isoforms and variant sequences, were obtained by filtering for “*M. musculus*” in the ‘model_organism’ field and selecting only reviewed records, and the “canonical and isoforms” download option. *C. albicans* SC5314 sequences were selected using the NCBI taxonomy identifier 237561, and again, both canonical and isoform sequences were downloaded. In silico trypsin digests were carried out using the “pepdigest” tool from the EMBOSS 6.6.0.0 package,^33^ assuming complete digestion and cutting only at favored sites (K/R not followed by K, R, I, F, L, or P) and a peptide length of >6 amino acids.

The resulting peptides were parsed using the custom Python code and pandas 1.5.3^34^ data frames constructed for both organisms. Four categories of peptide were identified using combinations of pandas concat(), unique(), and drop_duplicates() methods to create subsets of peptides:

(1) Unique within each organism, i.e., only occurring once within *M. musculus* or *C. albicans*
(2) Shared within each organism, i.e., peptides that map to multiple proteins within the same organism
(3) Unique to each organism
(4) Shared between the two organisms (referred to as cross-species peptides), i.e., peptides that map to proteins from both *M. musculus* and *C. albicans*

### Code Availability

All codes were produced in Jupyter notebooks and are freely available under an MIT license from https://github.com/bartongroup/-C.albicans-Peptide-Overlaps and interactively through MyBinder at https://mybinder.org/v2/gh/bartongroup/-C.albicans-Peptide-Overlaps/HEAD.

### FASTA Files

A *Mus musculus* SwissProt canonical with isoform (February 2022) database and *C. albicans* TrEMBL (May 2023) database were used for the Spectronaut searches.

### Data Availability

All raw files, Spectronaut sne files, Spectronaut reports, FASTA files, and the experimental template have been uploaded to PRIDE^35^ under accession number PXD045958 (https://www.ebi.ac.uk/pride/archive/projects/PXD045958).

## Results

### *M. musculus* and *C. albicans* Proteomes Contain Cross-Species Peptides

**Figure 1:**
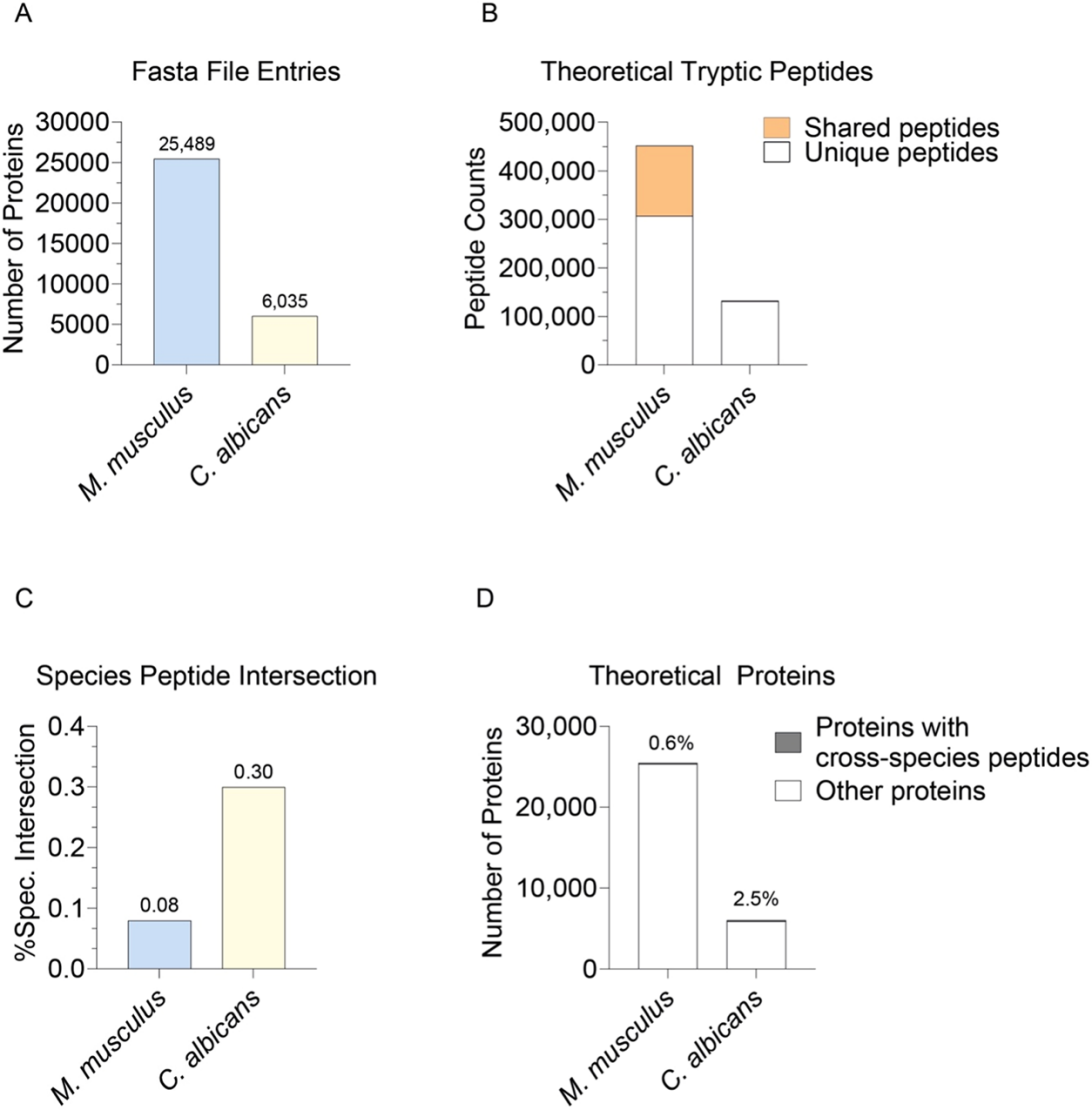
Homology between *M. musculus* and *C. albicans*: (A) Number of proteins present in the FASTA file entries for *M. musculus* and *C. albicans*. (B) Total number of theoretical tryptic peptide sequences found in *M. musculus* and *C. albicans* species. Shared peptides are highlighted in orange. Only peptides with 7 amino acids or more were considered. (C) The percentage of total cross-species peptides present in the *M. musculus* and *C. albicans* peptidome. (D) Number of proteins in and *C. albicans*. Proteins containing cross-species peptides are highlighted in gray.

To understand the theoretical homology between the host *Mus musculus* (*M. musculus*) proteome and the pathogenic *Candida albicans* (*C. albicans*) proteome, we performed an *in-silico* tryptic digest for both species (see Experimental Section). A comparison of their protein databases revealed that the *M. musculus* FASTA contained a total of 25,489 proteins, while the *C. albicans* FASTA contained 6,035 proteins (Figure 1A). An *in-silico* tryptic digest, which excluded peptides with less than 6 amino acids, resulted in 451,723 theoretical *M. musculus* peptides, 144,713 of which are shared between more than one *M. musculus* protein and 132,462 *C. albicans* peptide sequences, with 1,261 shared between more than one *C. albicans* protein (Figure 1B). We next sought to evaluate the number of peptides that were shared between *M. musculus* and *C. albicans*, referred to as cross-species peptides, to determine the potential for misidentifications. In total, there were only 351 cross-species peptides shared between *M. musculus* and *C. albicans*. This meant that only 0.08% of all *M. musculus* peptide sequences were shared with *C. albicans*, and 0.26% of all *C. albicans* peptides were shared with *M. musculus* (Figure 1C).

These cross-species peptides mapped to 148 *M. musculus* proteins, representing 0.6% of the entire *M. musculus* proteome (Figure 1D). As a result, when quantifying the *M. musculus* proteome via mass spectrometry in *C. albicans*-infected samples, there is limited potential for miss-assignment of *C. albicans* peptides as *M. musculus* peptides. This implies that only a small subset of *C. albicans* peptides could be wrongfully identified as *M. musculus* peptides and assigned to *M. musculus* proteins, hence adding quantitative noise.

### Including a FASTA File for Both Species Reduces Quantitative Disruptions

**Figure 2:**
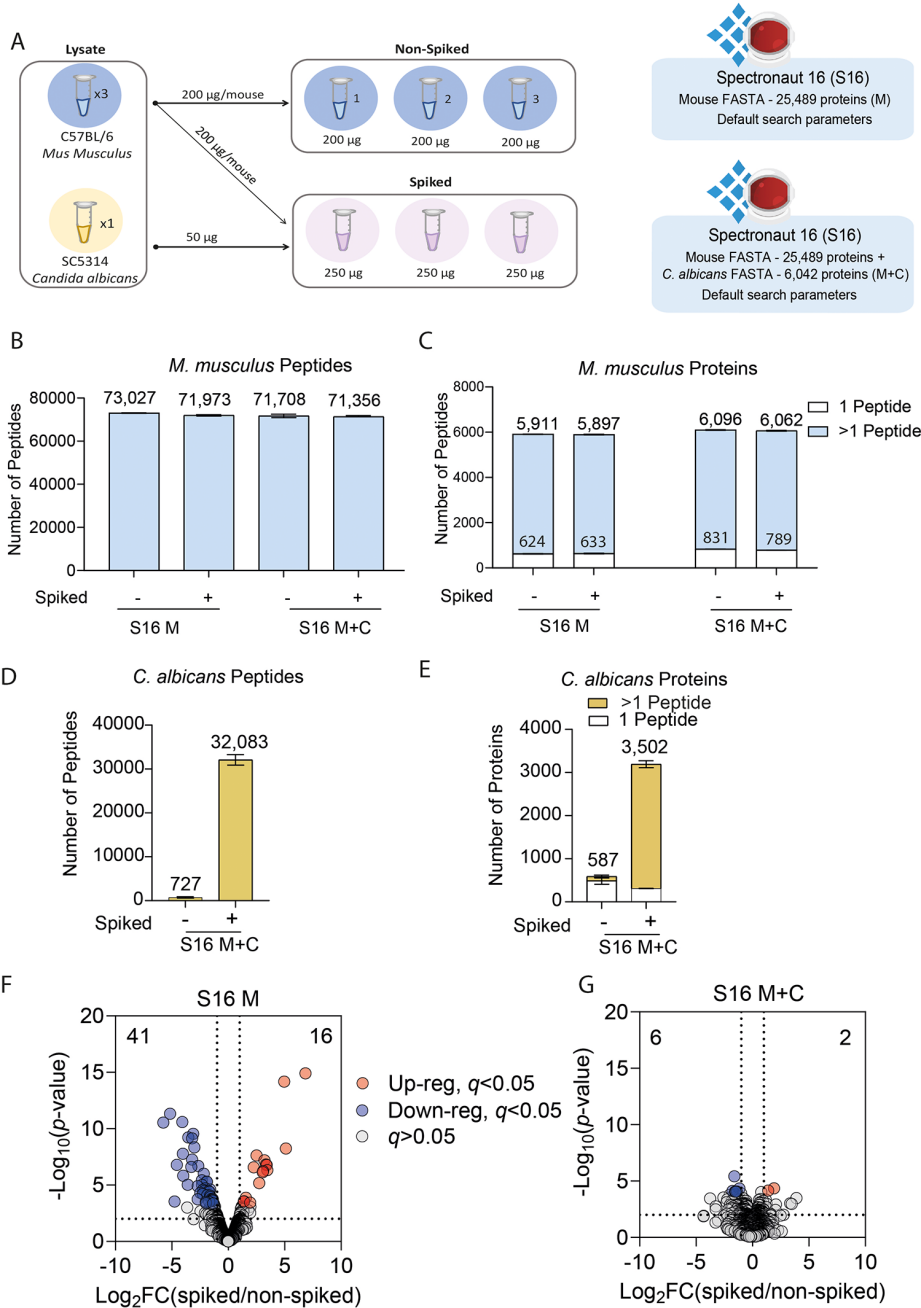
Identification and quantification errors: (A) Schematic of the experiment where *M. musculus* BMDMs and *C. albicans* were lysed separately for proteomics, and subsequently *C. albicans* lysates were spiked with the BMDM lysates (*M. musculusn* = 3, *C. albicansn* = 1). The number of (B) *M. musculus* peptides and (C) *M. musculus* proteins, identified using either a *M. musculus*-only (M) or *M. musculus* and *C. albicans* (M+C) FASTA. Proteins identified from only one peptide are highlighted in white. The number of (D) *C. albicans* peptides and (E) *C. albicans* proteins identified using the *M. musculus* and *C. albicans* (M+C) FASTA. Volcano plots showing the Log_2_ fold change of the spiked versus the nonspiked samples and the -Log_10_(*p*-value) for (F) Spectronaut 16 (S16) using default identification settings and a *M. musculus*-only FASTA and (G) Spectronaut 16 (S16) using default identification settings and a *M. musculus* and a *C. albicans* FASTA. Proteins with a *q*-value <0.05 and a log_2_ fold change >2 or <-2 are highlighted, as shown in the key.

To experimentally address whether the presence of *C. albicans* peptides affects the quantification of *M. musculus* proteins, we generated lysates from cultures of *M. musculus* bone marrow-derived macrophages (BMDMs) and separately *C. albicans* lysates. Three biological replicates of BMDMs were separated into two aliquots. One of the aliquots was spiked after lysis with *C. albicans* at a 1:4 ratio, referred to as “spiked”, and the other was left without any spike-ins, referred to as “nonspiked”. We then used a DIA-based analysis to characterize the proteome of the *M. musculus* BMDMs, comparing spiked and nonspiked samples (shown in Figure 2A), with the raw files being processed with Spectronaut.^8^

We first set out to determine whether it was beneficial to include both a FASTA file for *M. musculus* and a FASTA file for *C. albicans* within the search space. We found no major differences in the number of M. musculus peptides identified when using only a *M. musculus* FASTA or using both a *M. musculus* and a *C. albicans* FASTA file (Figure 2B). This also proved true when analyzing the number of *M. musculus* proteins identified (Figure 2C). We also checked the number of *C. albicans* peptides identified, and unsurprisingly, none were present if the FASTA was not included. We noticed, however, that when we included the *C. albicans* FASTA, we detected*C. albicans* peptides (Figure 2D) and proteins (Figure 2E) not only in the spiked but also the nonspiked samples, where they should not have been present.

We next set out to determine the quantitative differences of using only a *M. musculus* FASTA or both a *M. musculus* FASTA and a *C. albicans* FASTA. For this, we performed a differential expression analysis on the *M. musculus* proteome comparing the spiked and nonspiked samples. It is important to highlight that the *C. albicans* lysate was spiked after the BMDM samples were lysed; hence, no biological differences are expected between the spiked and nonspiked samples, though some technical differences can be present. The data showed that 57 proteins were significantly (see Experimental Section) changed between the spiked and nonspiked BMDMs when using only a *M. musculus* FASTA (Figure 2F). We inferred that this scenario was potentially caused by the assignment of the intensity of *C. albicans* peptides into *M. musculus* proteins. Interestingly, only 2 of these proteins contained a cross-species peptide between *M. musculus* and *C. albicans*, suggesting that there may also be an issue with incorrect peptide assignments. We hypothesized that also including the *C. albicans* FASTA file had the potential to improve the quantitative data and minimize the assignment of intensity from *C. albicans* peptides into *M. musculus* proteins. The addition of the *C. albicans* FASTA reduced the number of significantly changed proteins to 8 (Figure 2G). We determined that including the *C. albicans* FASTA file was important to minimize the quantitative errors. Hence, for all of the subsequent analyses, a *M. musculus* and a *C. albicans* FASTA were used.

### Optimizing Spectronaut Identification Settings Minimizes Misidentifications While Maintaining Proteome Depth

**Figure 3:**
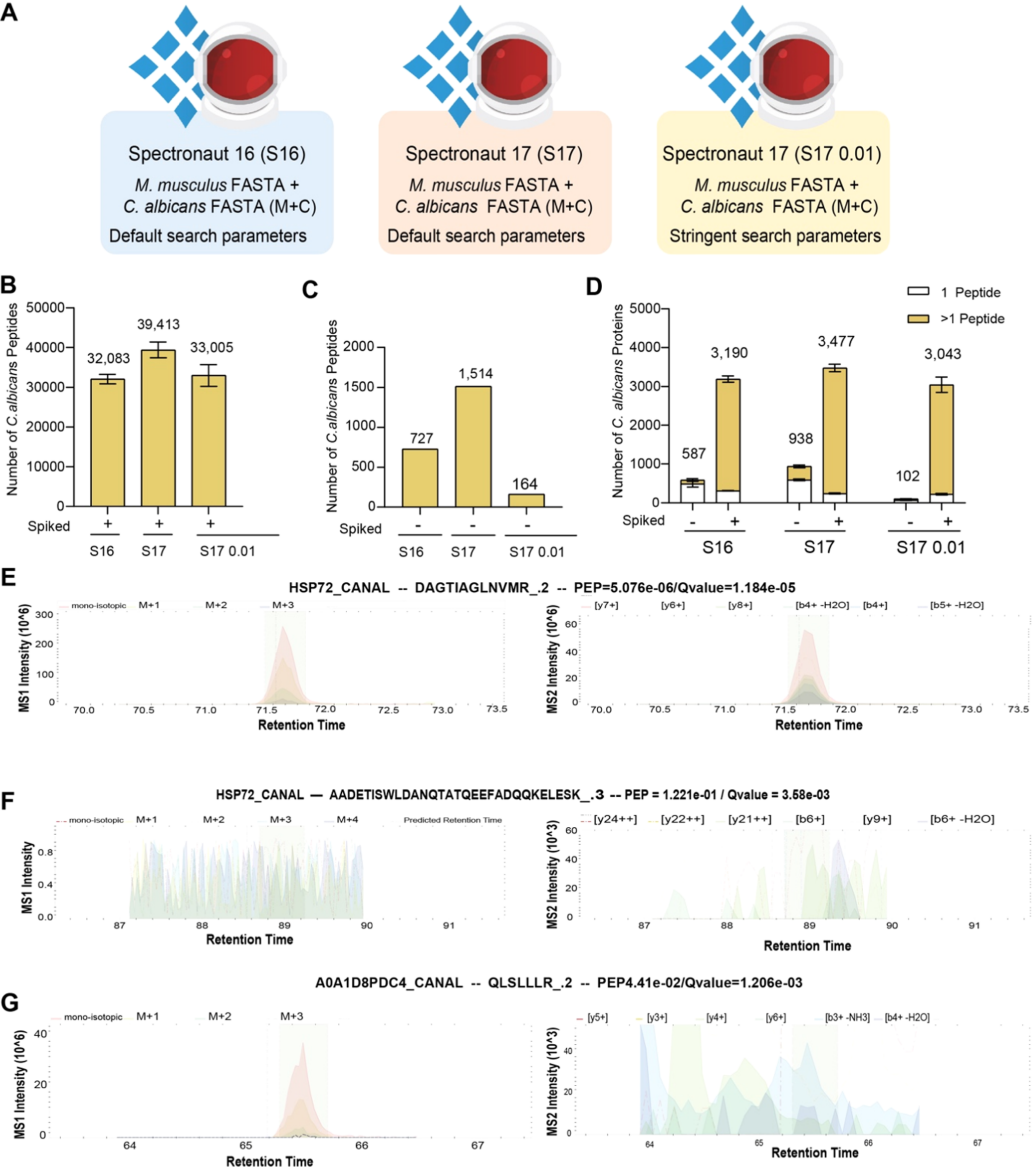
*C. albicans* peptide and protein identification across Spectronaut versions and settings: (A) Schematic of the data processing pipelines generated using Spectronaut 16 (S16) or 17 (S17) with default or stringent settings. The number of *C. albicans* peptides detected across the different pipelines in the (B) spiked samples and (C) nonspiked samples. (D) The number of *C. albicans* proteins detected in both spiked and nonspiked samples. Proteins identified with a single peptide are colored in white. Peptide (MS1) and precursor ion (MS2) eXtracted Ion Chromatogram (XIC) profiles for (E) protein HSP72_CANAL with precursor DAGTIAGLNMR_.2, (F) protein HSP72_CANAL with precursor AADETISWLDANQTATQEEFADQQKELESK_.3, and (G) protein A0A1D8PDC4_CANAL with precursor QLSLLLR_.2.

Having determined it was optimal to use both the*M. musculus* and *C. albicans* FASTA files (M+C), we focused on the potential false positives and misidentifications seen in the nonspiked samples (Figure 2D–E). For this work, we used Spectronaut (S) versions 16 and 17 (S16 or S17, respectively) (schematic of analysis in Figure 3A). In Spectronaut, the identification of peptides is determined by the peak properties and a target decoy approach. Therefore, a combined discriminant score is calculated for the peptide precursors and decoys based on machine learning.^36−38^ Then, the target and decoy distributions are fitted using a kernel density approach. These distributions are used to calculate the estimation of the run-wise false discovery rate (FDR)^36^ and posterior error probability (PEP). Additionally, a run-wise and experiment-wide protein FDR is estimated,^39^ and also, a PEP is calculated. These layers of FDR ensure that in large experiments, the overall protein FDR is estimated correctly. These settings can be easily tweaked on the interface and have been validated empirically using an entrapment approach using two species.^37^

We first compared both S16 and S17 by using the default settings. In this context, we focused our analysis on the number of *C. albicans* peptides and proteins that were identified both within the *C. albicans* spiked samples and the nonspiked samples. We first focused on the spiked samples. Here, S16 identified a total of 32,083 *C. albicans* peptides that matched to 3190 total proteins identified (Figure 3B–C). Those same samples analyzed with S17 using the same default settings identified 39,413 peptides, which represented a 20.5% increase, and these peptides mapped to 3477 proteins, which represented a 8.6% increase in the number of proteins identified. This suggests that S17 was the superior option, with respect to peptide and protein identification rates.

We next focused on *C. albicans* proteins and peptides that were detected in the nonspiked samples, where only *M. musculus* lysate was present. *C. albicans* proteins and peptides that were identified in the nonspiked samples provided examples of potential false positives. Here, we found that S16 identified 727 *C. albicans* peptides in the nonspiked samples, mapping to 587 proteins. S17 again displayed higher identification rates, with a mean of 1514 *C. albicans*peptides matched to 938 proteins in the nonspiked samples. Across all 3 replicates, a total of 1538 *C. albicans* proteins were identified in at least 1/3 replicates. We found only 94 out of the 1538 identified by S17 were cross-species peptides, suggesting that the majority of these peptides should not be present in the nonspiked samples and thus are considered to be false positives. This scenario is particularly problematic, as the false positives at the protein level represent ∼25% of the *C. albicans* proteome, which can lead to inaccurate biological interpretations.

We suspected that the previously seen false positives were caused by the erroneous transfer of identifications across the different runs, a similar situation is seen in DDA with “match between runs”.^40,41^ Hence, we decided to optimize the default search settings in Spectronaut to minimize the number of false positives. We increased the stringency of the default parameters by setting the posterior error probability to ≤0.01 and by setting the protein q-value scores across the runs to ≤0.01 (analysis settings denoted S17 0.01; see Experimental Section).

Our data showed that the updated parameters were extremely effective in removing false identifications in the nonspiked samples, reducing the mean number of *C. albicans* peptides detected from 1514 to 164, and the mean number of *C. albicans* proteins from 938 to 102. Across all 3 nonspiked samples, a total of 164 *C. albicans* proteins were detected; 89 of which contained cross-species peptides. This suggested that for 54% of these proteins, the issues were not caused by spurious peptide identifications but by incorrect peptide-to-protein group assignment. The data also revealed that the majority of *C. albicans* peptides that were still detected with the optimized parameters had clear extracted ion chromatogram (XIC) peaks at the predicted retention times (Figure 3E and Supporting Information Figures 1–2), whereas the identifications that were filtered out frequently displayed no concrete precursor ion XIC peaks (Figure 3F–G and Supporting Information Figures 3–4 for each replicate spectra).

**Figure 4:**
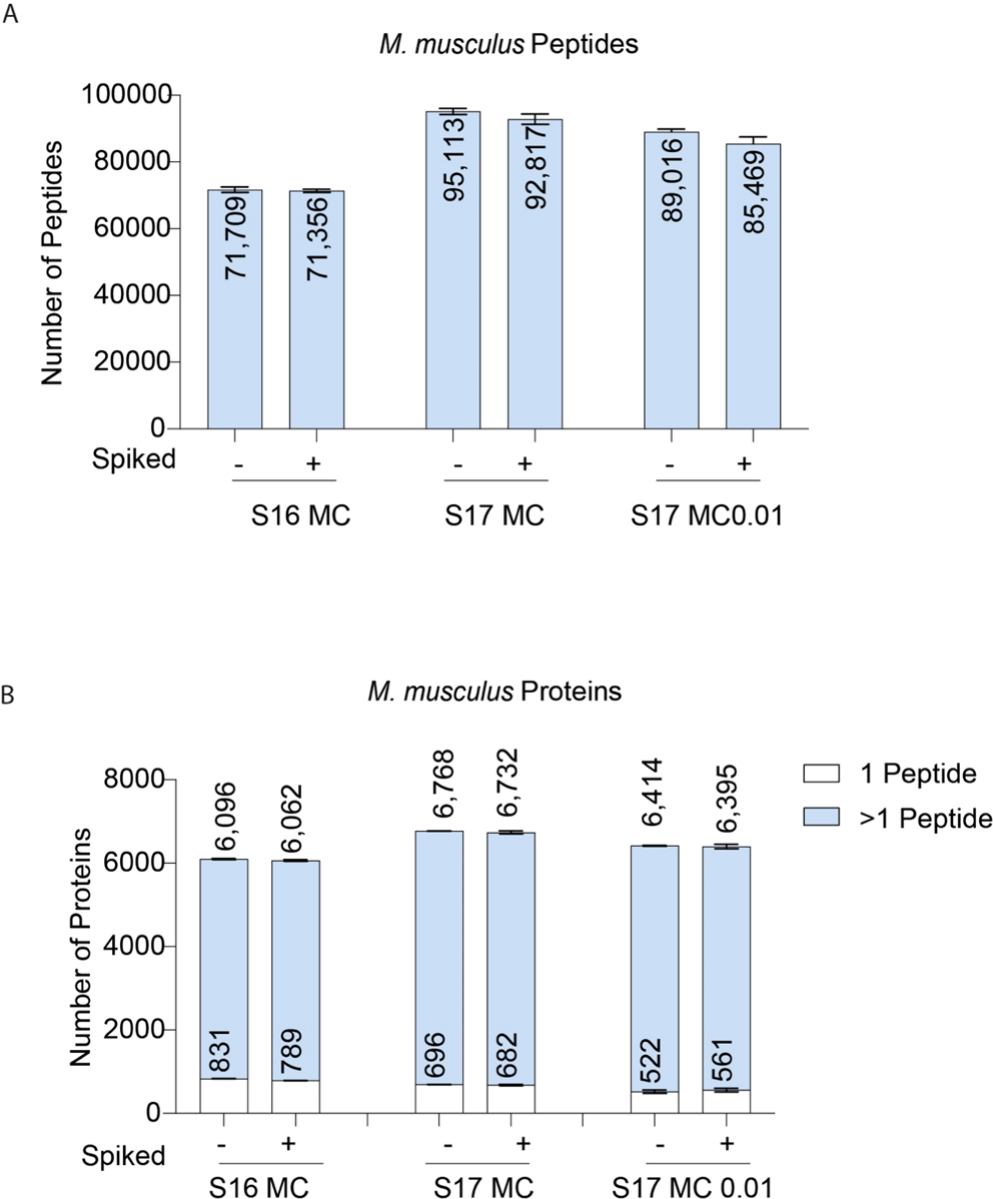
*M. musculus* identifications across Spectronaut settings: Number of (A)*M. musculus* peptides and (B) *M. musculus* proteins identified in spiked and nonspiked samples. Proteins identified with one peptide are noted in white.

We also wanted to understand the effect that the stringent parameters would have on *M. musculus* proteome coverage, i.e., *M. musculus* peptides found in the nonspiked samples, as we suspected that the stringent parameters might cause a considerable reduction in the number of these peptides. The standard setting on S17 led to the identification of 95,113 *M. musculus* peptides and 6,768 proteins in the nonspiked samples (Figure 4A,B). The use of the stringent settings only led to a reduction of 6.4% in the number of *M. musculus* peptides identified (89,016 peptides) in this sample, which translated into a 5.2% reduction in the number of *M. musculus* proteins (6,414 proteins) identified. Furthermore, the number of *M. musculus* proteins identified with the stringent parameters in S17 was still higher than the standard parameters on S16, suggesting that these optimized settings on the newer versions of Spectronaut are a very effective strategy to obtain increased data quality at a small penalty. Additionally, the quantification of *M. musculus* proteins highlighted as significantly changed due to the presence of *C. albicans*, of which just one contained cross-species peptides, was reduced by >98% in S17 0.01, while still being detected within the data set (Supporting Information Figure 5).

Overall, although we did not entirely remove misidentifications with our optimized settings, we were able to make significant improvements without a considerable reduction of proteome depth.

## Discussion

DIA-based proteomic workflows are characterized by having much higher complexity spectra than what is usually encountered in the narrow DDA isolation window.^3−5^ The computational solutions to analyze such complex data originally required specific peptide or spectral libraries to be generated.^6−8^ These libraries were generated in DDA and added cost and complexity to the DIA projects. A big breakthrough toward the widespread implementation of DIA occurred with the implementation of library-free searches across different software tools.^42−44^ This meant that it was no longer necessary to generate expensive DDA-based libraries. However, the complexity of the spectra is still present, and though library-free search algorithms leverage deep neural networks to improve the matching and deconvolution,^37,45^ some issues remain.

In immunological research, it is not uncommon to study infection models, where a host, i.e., *M. musculus*, is infected by a pathogen, such as *C. albicans*.^20,21^ Hence, we became interested in understanding the potential complications that could arise when analyzing these two proteomes within a single sample. This scenario represents two heterogeneous populations, where some proteins should only be present in one of the two populations, which frequently occurs in proteomic studies. Here, we compared the proteomes of *M. musculus* macrophages that were either spiked with *C. albicans* postlysis or not spiked.

*M. musculus* and *C. albicans* displayed a low level of sequence homology, which suggested limited issues related to peptide misidentifications across the two species. However, our data revealed that using the default search settings, 1,514 peptides and 938 proteins were identified in the nonspiked murine samples, representing ∼25% of the *C. albicans* proteome. All of the 1,514 peptides identified in the nonspiked samples were detected in the*C. albicans* spiked samples, the majority with robust PEPs and XIC peaks. This suggests that the peptides were correctly identified in those spiked samples. The issue with the *C. albicans* peptides detected in the nonspiked samples centered around the transfer of these identifications across the different runs, an issue that has been described in DDA with “match between runs” algorithms.^40,41,46^

Thus, we optimized the search parameters to increase the stringency. First, we set the protein q-value cutoff per run to <0.01, this would increase the strictness of the identification transfers across the different samples. Second, we set the posterior error probability (PEP) at the precursor and protein level to <0.01, increasing the stringency of the overall identifications. These updated parameters proved extremely successful in improving the quality of the identifications, reducing the number of false positive identifications of *C. albicans* peptides and proteins present in the nonspiked samples by ∼90%. Importantly, this increased stringency came at a limited cost, where the total *M. musculus* peptide identifications, which we will call true positives, were only reduced by 6.4%. We believe our work is not limited to multispecies but would also be of great value when analyzing heterogeneous populations within the same experiment. For example, a DIA study looking at multiple distinct cell types or studying different tissues would also greatly benefit from the previously mentioned reduction in false positives.

As newer instruments and analytical methods enable a more comprehensive coverage while using shorter gradients,^1,2,9^ we believe the focus should migrate from maximizing the number of identifications to increasing the quality of such identifications. We show that a way to achieve this when using Spectronaut requires modifying only a small subset of search parameters, which results in dramatically reduced false positives with only a minor reduction in overall proteome depth.

It is also worth noting that our work manually selected a subset of peptides and fragment ions, which we knew should be absent from specific populations. This process discovered matches without discernible XIC peaks. It be of great benefit to develop a tool that systematically analyzed the peptide and precursor identifications, flagging up the number of peptides and precursor ions without discernible XIC peaks within the expected retention times. If this is integrated into an automated reporting tool, it will enable a much easier and user-friendly approach to monitor DIA data quality.

## Data Availability

All raw files, Spectronaut sne files, Spectronaut reports, FASTA files, and the experimental template have been uploaded to PRIDE^35^ under accession number PXD045958 (https://www.ebi.ac.uk/pride/archive/projects/PXD045958).

## Supporting Information Available

XIC plot for DAGTIAGLNVMR_.2 with standard settings (Figure S1); XIC plot for DAGTIAGLNVMR_.2 with stringent settings (Figure S2); XIC plot for AADETISWLDANQTATQEEFADQKELESK_.3 (Figure S3); XIC plot for MSSHIFDLK_.1 (Figure S4); significantly changed proteins across the different Spectronaut settings (Figure S5) (PDF)
