# Supplementary material for: Optimizing Spectronaut Search Parameters to Improve Data Quality with Minimal Proteome Coverage Reductions in DIA Analyses of Heterogeneous Samples
Source: J Proteome Res. 2024 May 1;23(6):1926–36. doi: 10.1021/acs.jproteome.3c00671 (PMC11165578; doi:10.1021/acs.jproteome.3c00671)
Supplement: Supplementary file 1 — pr3c00671_si_001.pdf [file pr3c00671_si_001.pdf]

Supplementary data for

Optimising Spectronaut search parameters to improve data quality with minimal proteome coverage reductions in DIA analyses of heterogeneous samples

Christa P. Baker[1], Roland Bruderer[2], James Abbott[3], J. Simon C. Arthur[1*], Alejandro J. Brenes[1,4*]

Affiliations:

1- Division of Cell Signalling & Immunology, School of Life Sciences, University of Dundee, United Kingdom

2- Biognosys AG, Schlieren, Switzerland

3- Data Analysis Group, Division of Computational Biology, School of Life Sciences, University of Dundee, United Kingdom

4- Present address: Centre for Inflammation Research, Institute for Regeneration and Repair, University of Edinburgh, EH16 4UU, United Kingdom

* correspondence: Alejandro J. Brenes, abrenes@ed.ac.uk, Simon Arthur, j.s.c.arthur@dundee.ac.uk

Table of contents

Supplemental Figure 1: XIC for DAGTIAGLNVMR_.2 with standard settings

Supplemental Figure 2: XIC for DAGTIAGLNVMR_.2 with stringent settings

Supplemental Figure 3: XIC for AADETISWLDANQTATQEEFADQKELESK_.3

Supplemental Figure 4: XIC for MSSHIFDLK_.1

Supplemental Figure 5: Significantly changed proteins across the different Spectronaut settings

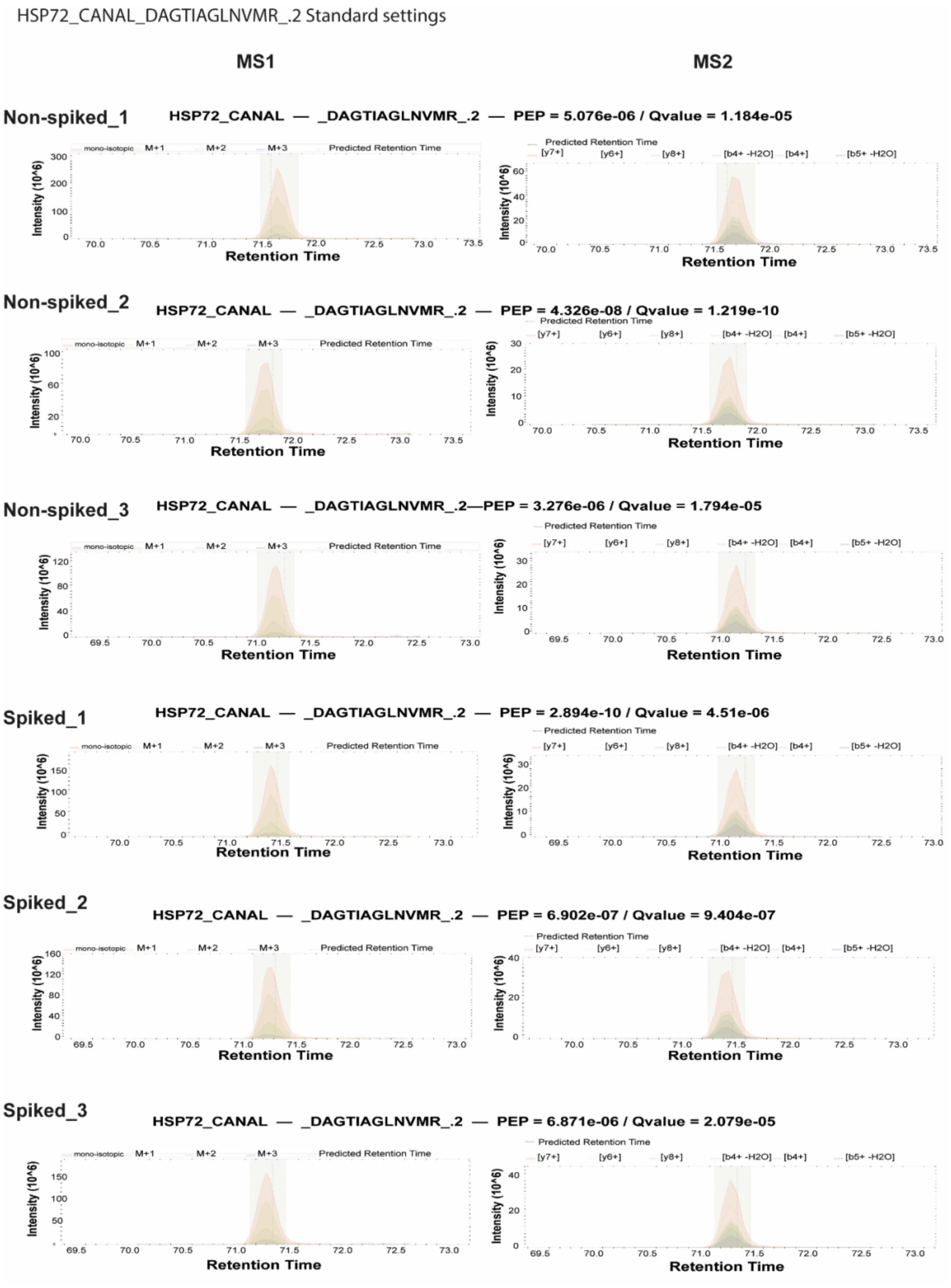

Supplementary Figure 1: Spectronaut 17 default settings MSI and MS2 extracted ion chromatogram (XIC) profiles for *C. albicans* protein HSP72 and precursor DAGTIAGLNVMR_.2, across all samples, both spiked and non-spiked.

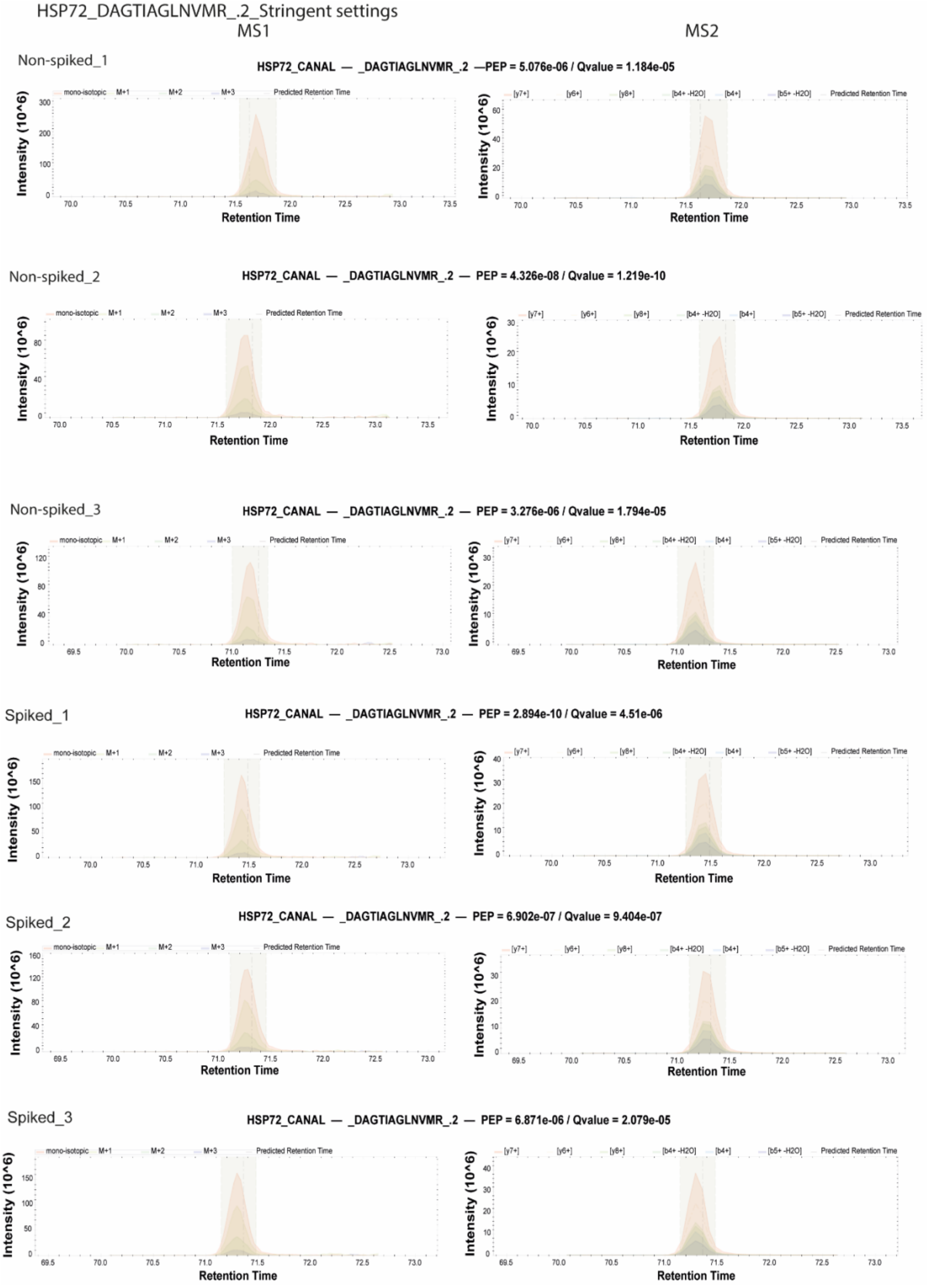

Supplementary Figure 2: Spectronaut 17 stringent settings MSI and MS2 extracted ion chromatogram (XIC) profiles for *C. albicans* protein HSP72 and precursor DAGTIAGLNVMR_.2, across all samples, both spiked and non-spiked.

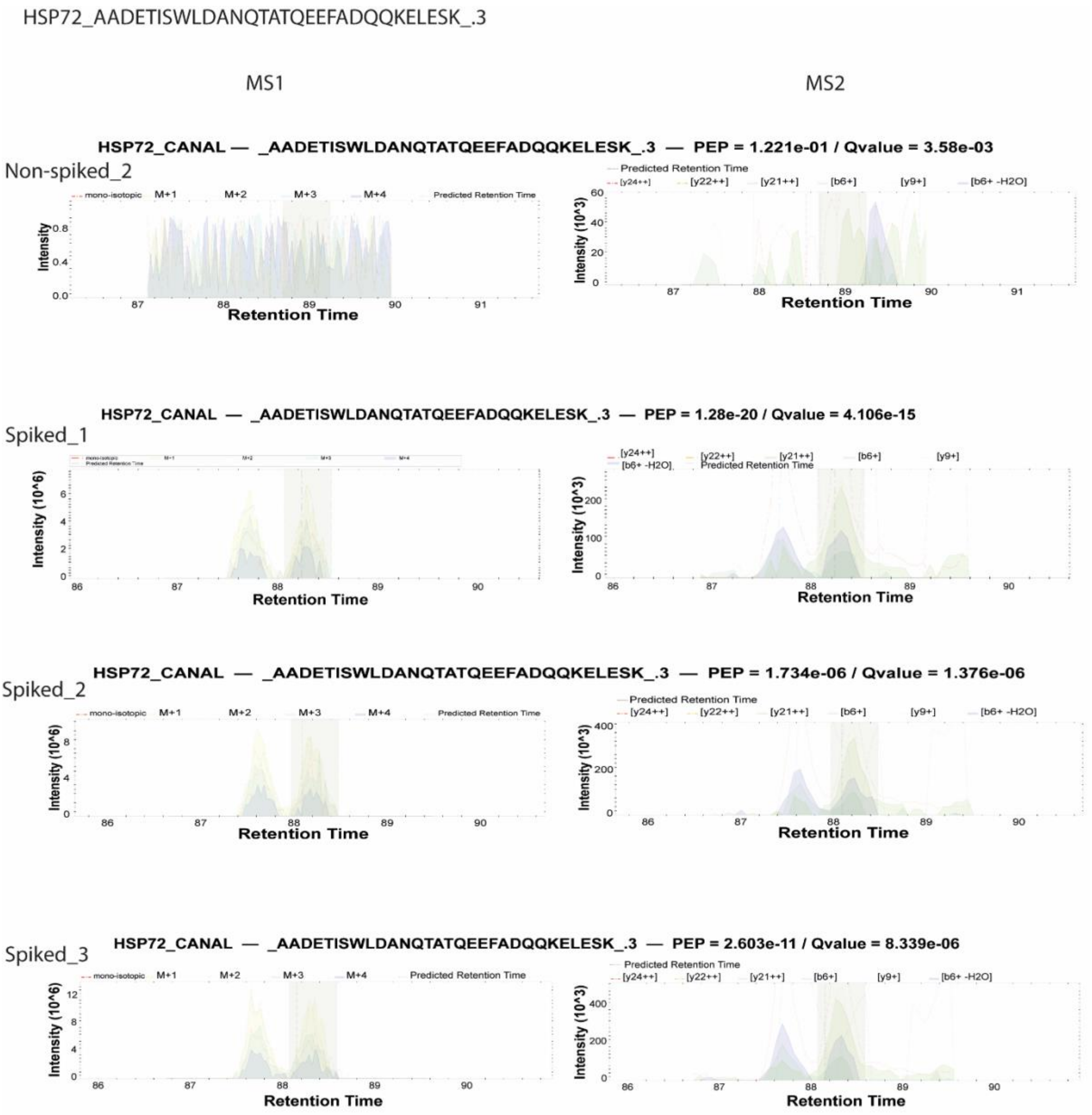

Supplementary Figure 3: Spectronaut 17 default settings MSI and MS2 extracted ion chromatogram (XIC) profiles for *C. albicans* protein HSP72 and precursor AADETISWLDANQTATQEEFADQQKELESK_.3, across all samples, both spiked and non-spiked.

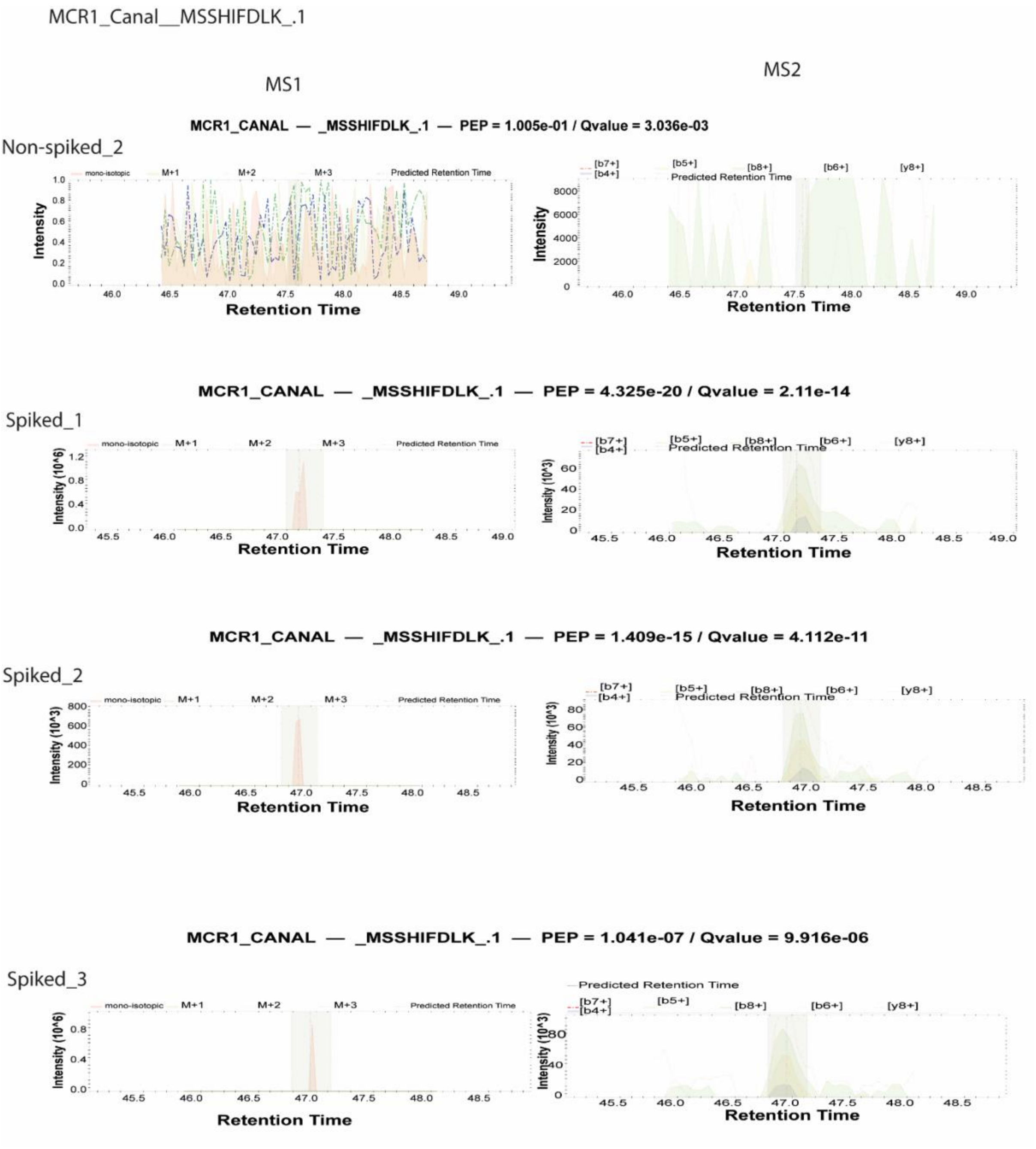

Supplementary Figure 4: Spectronaut 17 default settings MSI and MS2 extracted ion chromatogram (XIC) profiles for *C. albicans* protein HSP72 and precursor MSSHIFDLK_.1, across all samples, both spiked and non-spiked.

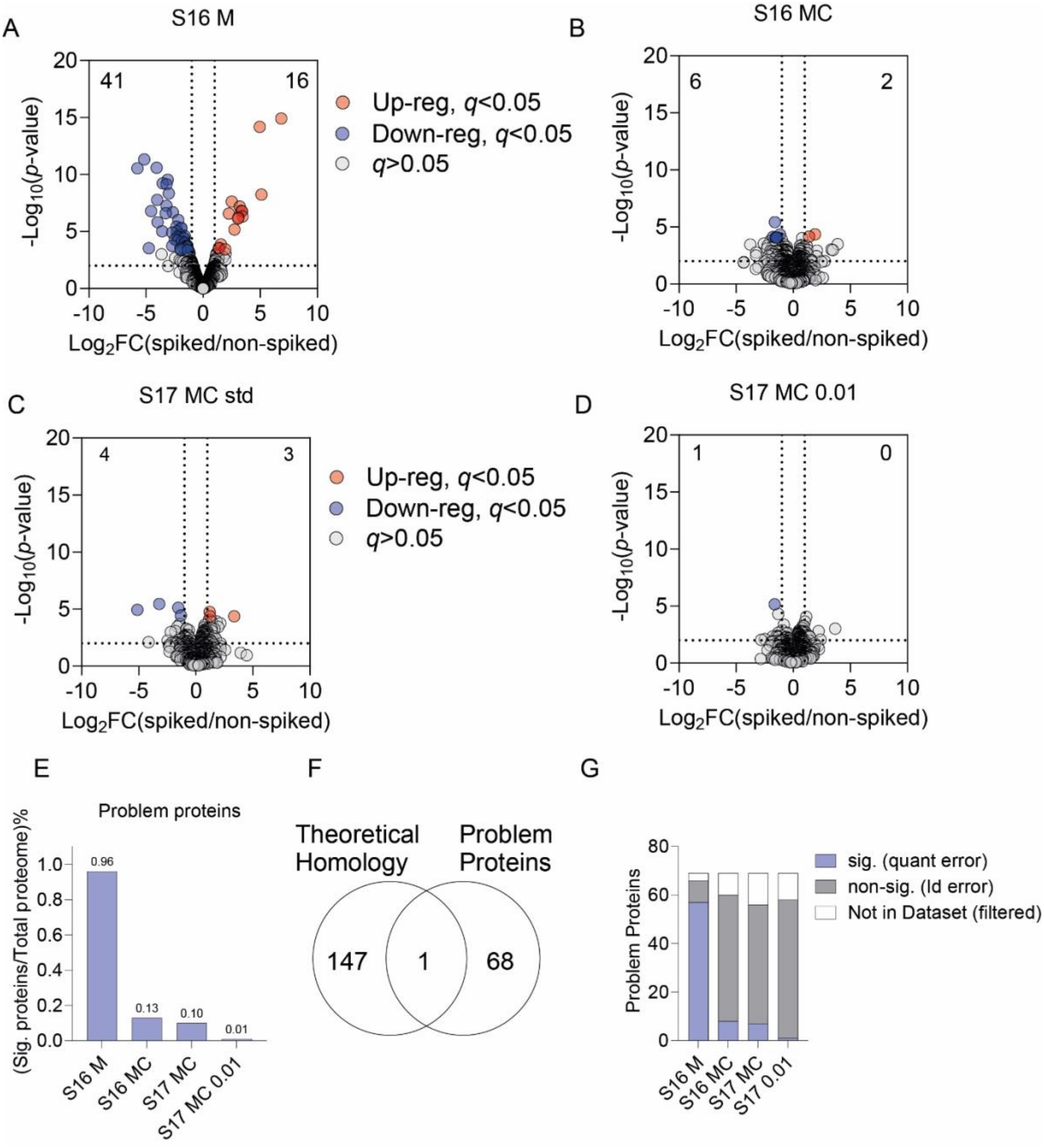

Supplementary Figure 5. Volcano plots for (**A)** Spectronaut 16, default settings with a Mouse only FASTA file, and **(B)** Spectronaut 16, default settings **(C)** Spectronaut 17, default settings and **(D)** Spectronaut 17 with 0.01 identification settings with a Mouse FASTA and a Candida FASTA. (D-E) The significant proteins from each analysis were pooled to make a compiled list of "problematic proteins" q-value <0.05 and fold change >2. **(E)** Plots problematic proteins in each analysis as a percentage of the total proteins identified in the dataset. **(F)** compares this list of Mouse problematic proteins against the theoretical homology between Mouse and *C. albicans* to determine how many of these errors are due to homology or misidentification while **(G)** plots the problematic proteins, highlighting the number of significant or non-significant proteins for that analysis, as well as proteins that were not identified in the analysis. Volcano plots show the Log_2 fold change (spiked vs non-spiked), and the $-Log_{10}$ p-value and highlights proteins with a p-value< 0.01 and -2>FC>2.
